# Net Clinical Benefit of Oral Anticoagulants: A Multiple Criteria Decision Analysis

**DOI:** 10.1371/journal.pone.0124806

**Published:** 2015-04-21

**Authors:** Jason C. Hsu, Cheng-Yang Hsieh, Yea-Huei Kao Yang, Christine Y. Lu

**Affiliations:** 1 School of Pharmacy and Institute of Clinical Pharmacy and Pharmaceutical Sciences, National Cheng Kung University, Tainan, Taiwan; 2 Stroke Center and Department of Neurology, Tainan Sin Lau Hospital, Tainan, Taiwan; 3 Department of Population Medicine, Harvard Medical School and Harvard Pilgrim Health Care Institute, MA, United States of America; US Army Engineer Research and Development Center, UNITED STATES

## Abstract

**Background:**

This study quantitatively evaluated the comparative efficacy and safety of new oral anticoagulants (dabigatran, rivaroxaban, and apizaban) and warfarin for treatment of nonvalvular atrial fibrillation. We also compared these agents under different scenarios, including population with high risk of stroke and for primary vs. secondary stroke prevention.

**Methods:**

We used multiple criteria decision analysis (MCDA) to assess the benefit-risk of these medications. Our MCDA models contained criteria for benefits (prevention of ischemic stroke and systemic embolism) and risks (intracranial and extracranial bleeding). We calculated a performance score for each drug accounting for benefits and risks in comparison to treatment alternatives.

**Results:**

Overall, new agents had higher performance scores than warfarin; in order of performance scores: dabigatran 150 mg (0.529), rivaroxaban (0.462), apixaban (0.426), and warfarin (0.191). For patients at a higher risk of stroke (CHADS_2_ score≥3), apixaban had the highest performance score (0.686); performance scores for other drugs were 0.462 for dabigatran 150 mg, 0.392 for dabigatran 110 mg, 0.271 for rivaroxaban, and 0.116 for warfarin. Dabigatran 150 mg had the highest performance score for primary stroke prevention, while dabigatran 110 mg had the highest performance score for secondary prevention.

**Conclusions:**

Our results suggest that new oral anticoagulants might be preferred over warfarin. Selecting appropriate medicines according to the patient’s condition based on information from an integrated benefit-risk assessment of treatment options is crucial to achieve optimal clinical outcomes.

## Introduction

The prevalence and incidence of atrial fibrillation (AF) have increased in the past two decades, in part due to the aging population [1]. More than 6.5 million patients are diagnosed with AF in the US currently, and the number is estimated to reach 12.1 million in 2050 [2–3]. AF is a major risk factor for ischemic stroke [3] and accounts for about 12–20% of ischemic strokes [4–5]. The importance of active prevention of AF-induced stroke is increasingly recognized.

Warfarin has been used to lower the risk of stroke in patients with AF for decades. Previous studies indicated that dose-adjusted warfarin significantly reduces the incidence of stroke, but its use is associated with risk of bleeding [6–7]. Patients on warfarin therapy must be periodically monitored with dose adjustments as necessary. In recent years, new oral anticoagulants (dabigatran, rivaroxaban, and apizaban) became available as treatment options for preventing stroke for patients with AF [8]. These medicines have the characteristics of ideal oral anticoagulants for long-term use: effective in preventing stroke, low risk of bleeding, a wide therapeutic window, a low propensity for food and drug interactions, and administration in fixed dosages [6, 8]. The 2011 guideline by American Heart Association recommended using dabigatran as the alternative in patients with AF [9].

A number of large randomized controlled trials have shown the efficacy and safety of new oral anticoagulants in patients with AF. These include the RE-LY trial [10–11], ROKET-AF trial [12], AVERROES trial [13–14], and ARISTOTLE trial [14]. In the last few years, several meta-analyses have been conducted to study the comparative efficacy and safety of oral anticoagulants [8, 15–19]. They found no statistically significant difference in efficacy (e.g., prevention of ischemic stroke) between the three drugs, although apixaban and dabigatran were numerically superior to rivaroxaban [16–17]. However, with a lower risk of intracranial bleeding, new oral anticoagulants appear to have a more favorable safety profile, making them promising alternatives to warfarin [8]. Particularly, apixaban produced significantly fewer major bleedings [16–17] and gastrointestinal bleedings [20] than dabigatran 150 mg twice a day (BID) and rivaroxaban 20 mg once a **day** (QD). Dabigatran 150 mg BID was superior to rivaroxaban for some efficacy endpoints, whereas major bleeding was significantly lower with dabigatran 110 mg BID and apixaban 5 mg BID [17].

Despite these studies, it remains unclear which new anticoagulant agent is the optimal choice. There is a lack of integrated benefit-risk assessment of treatment options. Thus, evidence is limited in terms of the benefit-risk balance for each oral anticoagulant, for each clinical endpoint representing risks or benefits, and for patients of different characteristics [21].

A recent analysis by Canestaro et al. (2013) assessed the cost-effectiveness of oral anticoagulants for treatment of AF among patients aged 70 years with an average CHADS_2_ score, a clinical prediction rule for estimating the risk of stroke in patients with non-rheumatic AF [22–23]. This analysis used a Markov state transition decision tree model, a threshold of $100,000 per quality-adjusted life year, and incremental cost-effectiveness ratios [24]. However, instead of providing a priority/ranking of medications for decision making, it merely used “incremental costs” to express the additional financial burden needed to bear under specific conditions in order to achieve the similar treatment effect (with consideration of risk) between alternative drugs. Further, rather than comparing drugs for specific clinical endpoints representing risks and benefits (e.g., intracranial bleeding), it merely assessed broadly defined outcomes (e.g., bleeding). Lastly, this study focused on the general scenario (70-year-old patients with an average CHADS_2_ score) only without considering other common treatment scenarios.

Our study aims to address the above gaps by using multiple criteria decision analysis (MCDA) to conjointly and quantitatively evaluate the comparative efficacy and safety of new oral anticoagulant agents and warfarin for treatment of nonvalvular atrial fibrillation. We also compared these agents under different scenarios: general scenario, patients with a high risk of stroke (CHADS_2_ score≥3), and patients taking one of these medicines for primary or for secondary stroke prevention. We chose this method because it can evaluate therapeutic alternatives by integrating multiple criteria in an explicit manner, including the trade-offs between benefits and risks [25–26].

## Method

MCDA is a method for supporting decision making and has been used in fields of economics and management [27–28]. MCDA can also be used to support healthcare decisions, for instance, for assessing risks and benefits of medical technologies. It is a sound framework for integrating multiple benefit and risk criteria based on their perceived value, and using this as a basis for comparing alternative treatments. Its advantages, compared with head-to-head clinical trials, include expressing all effects (favorable and unfavorable) of different performance metrics (e.g., measurable quantities, scoring systems, relative frequencies, health outcomes); capturing trade-offs among the effects; and calculating the weight for each outcome against other outcomes [25–26]. Using a decision value tree, a performance score can be calculated for a drug to represent its benefit-risk balance, considering the health utilities, relative to other drugs. Both benefit and risk criteria can be split into multiple sub-criteria in case of different primary endpoints, subgroups, and interactions [21, 26, 29]. We used similar methods to assess the comparative benefit-risk balance of oral phosphodiesterase type 5 inhibitors for erectile dysfunction [30].

Decision models were created to compare the benefit-risk balance of new oral anticoagulant agents and warfarin under four scenarios: (1) the general population (70 year-old), (2) patients with a higher risk of stroke (CHADS_2_ score≥3), (3) primary stroke prevention, and (4) secondary stroke prevention. Selection of criteria and sub-criteria in this study was based on good decision analysis practice [31–32], which recommends that criteria should have the following key characteristics: being clinically important, essential, non-redundant, operational, complete, and reflecting the goals of decision and questions of study. According to these principles, we reviewed the literature and selected criteria (benefits and risks) to create our decision models.

**Fig 1:**
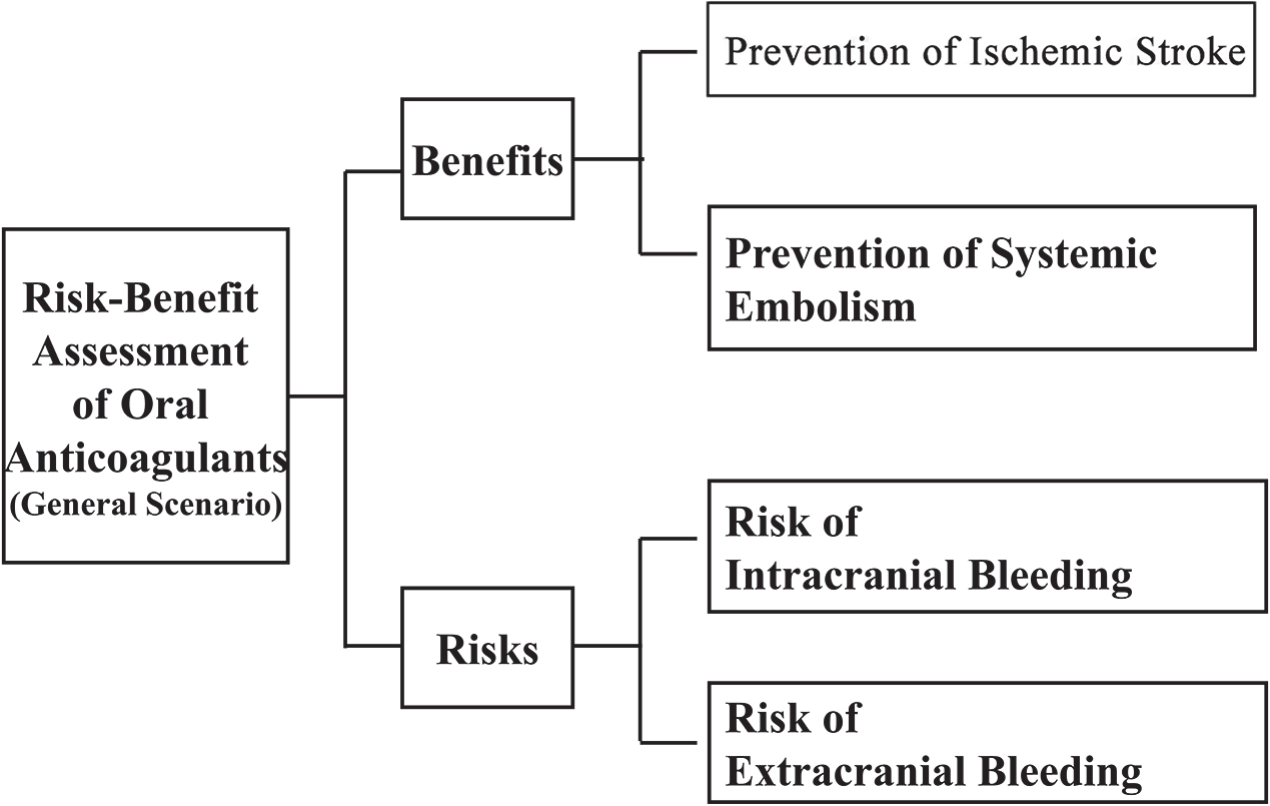
Decision model.

For the general population, we followed Canestaro et al (2013) [24] and used the effect sizes of prevention of ischemic stroke and prevention of systemic embolism as sub-criteria for benefits, and risks of intracranial bleeding and extracranial bleeding as sub-criteria for risks (Fig 1). For patients with a higher risk of stroke, we followed Schneeweiss et al (2012) [16] and used prevention of stroke or systemic embolism as a sub-criterion for benefits, and major bleeding as a sub-criterion for risks. For primary or secondary stroke prevention, we followed Rasmussen et al (2012) [15] and used prevention of stroke or systemic embolism and prevention of death from vascular causes as sub-criteria for benefits, and intracranial bleeding and other local bleeding as sub-criteria for risks. These three studies were published in major journals and their data sources were large RCTs and/or meta-analyses of RCTs. Therefore, we selected these studies to provide the comparative data on benefits (the reciprocal of hazard ratios to represent “prevention” effects) and risks (hazard ratios) of oral anticoagulants and warfarin on various sub-criteria for our models, which are not available from single RCTs, or cohort or case-control studies [15–16, 24]. Warfarin was used as a baseline alternative when it is one of the options; otherwise, rivaroxaban was used as a baseline alternative.

Our MCDA modeling overall involved the following steps: (i) develop decision models with multiple criteria (decision value tree), (ii) pull data from the literature on effect sizes (with 95% confidence intervals) for benefit and risk criteria for each drug compared with other drugs, (iii) for each criterion, scale and normalize the values of the effect (and 95% confidence intervals) of all drugs so that the range is between 0 and 1, (iv) estimate the weight for each criterion against other criteria based on health utilities representing their relative importance from patients’ perspectives, (v) and finally based on ranking of drugs using standardized effect sizes and weighting of their health utilities, calculate a performance score for each drug, integrating its benefit-risk balance, considering health utilities, against therapeutic alternatives.

To accommodate uncertainties, instead of inputting only the exact values for hazard ratios (or the reciprocal of hazard ratios) reported in published studies, we used the “Three Point Estimate” method to allow the model to consider values within the 95% confidence interval of the hazard ratios (or the reciprocal of hazard ratios). Table 1 shows the data imputed in the model, including the overall and different scenarios [15–16, 24, 33–35].

**Table 1:** Treatment effects and risks inputted in models.

Criteria	Scenario	Criteria	Units	Oral Anticoagulant agents					Reference
				Warfarin	Dabigatran 150 mg BID	Dabigatran 110 mg BID	Rivaroxaban 20 mg QD	Apixaban 5 mg BID	
Benefit	General (70 year old)	Prevention of ischemic stroke	1/HR	1	1.32 (1.02–1.67)		1.06 (0.85–1.33)	1.09 (0.89–1.35)	24
		Prevention of systemic embolism	1/HR	1	1.20 (0.93–1.72)		4.35 (1.64–11.11)	1.15 (0.57–2.27)	24
	High risk of stroke	Prevention of stroke or systemic embolism	1/HR	1	1.43 (1.05–1.92)	1.27 (0.95–1.69)	1.14 (0.95–1.35)	1.47 (1.14–1.92)	16
	Primary stoke prevention	Prevention of stroke or systemic embolism	1/HR		1.28 (0.87–1.89)	0.83 (0.57–1.19)	1	0.94 (0.65–1.35)	15
		Prevention of death from vascular causes	1/HR		1.11 (0.85–1.47)	0.91 (0.69–1.19)	1	1.02 (0.79–1.33)	15
	Secondary stroke prevention	Prevention of stroke or systemic embolism	1/HR		1.25 (0.83–1.92)	1.12 (0.74–1.69)	1	1.23 (0.85–1.79)	15
		Prevention of death from vascular causes	1/HR		1.00 (0.68–1.47)	1.56 (1.01–2.38)	1	1.00 (0.68–1.45)	15
Risk	General (70 year old)	Risk of intracranial bleeding	HR	1	0.40 (0.27–0.60)		0.67 (0.47–0.93)	0.42 (0.30–0.58)	24
		Risk of extracranial bleeding	HR	1	1.07 (0.92–1.25)		0.42 (0.29–0.55)	0.79 (0.68–0.93)	24
	High risk of stroke	Risk of major bleeding	HR	1	1.05 (0.86–1.30)	0.82 (0.66–1.03)	1.01 (0.87–1.18)	0.69 (0.55–0.87)	16
	Primary stoke prevention	Risk of intracranial bleeding	HR		0.75 (0.38–1.52)	0.61 (0.30–1.27)	1	0.77 (0.40–1.49)	15
		Risk of other local bleeding	HR		1.67 (0.76–3.70)	1.57 (0.71–3.48)	1	1.23 (0.55–2.72)	15
	Secondary stroke prevention	Risk of intracranial bleeding	HR		0.55 (0.25–1.23)	0.27 (0.10–0.73)	1	0.50 (0.24–1.04)	15
		Risk of other local bleeding	HR		2.56 (1.12–5.88)	1.74 (0.75–4.04)	1	1.92 (0.83–4.46)	15

High risk of stroke: patients with CHADS_2_ score ≥3.

HR = hazard ration; 1/HR = the reciprocal of hazard ratio.

QD: Once a day; BID: twice a day.

To compare drugs for each single criterion, we scaled and normalized the values of the effect and their intervals using “Single-attribute Utility Function” [36] method with appropriate quadratic function so that the range is between 0 and 1 (0 represents the least preferred and reflects the lowest possible value among all drugs; and 1 represents the most preferred and reflects the highest possible value among all drugs). The quadratic utility functions are defined as follows:

- for benefit criteria:
  $$\begin{array}{l} u(x)=ax^{2}+bx+c;\;u(\min)=0;\;u(\max)=1;\;u\left\{\min+\left[\left(\max-\min\right)/2\right]\right\}=0.75\\ u(x)=2\left[\left(x-\min\right)/\left(\max-\min\right)\right]-{\left[\left(x-\min\right)/\left(\max-\min\right)\right]}^{2} \end{array}$$
- for risk criteria:
  $$\begin{array}{l} u(x)=ax^{2}+bx+c;\;u(\min)=1;\;u(\max)=0;\;u\left\{\min+\left[\left(\max-\min\right)/2\right]\right\}=0.25\\ u(x)={\left[\left(x-\min\right)/\left(\max-\min\right)\right]}^{2} \end{array}$$

Next, we estimated the weight for each criterion against other criteria based on health utilities representing their relative importance that partly reflect the severity of disease and patients’ concerns. We reviewed the literature for existing health utility measures (mean and range from experts’ and patients’ views) of all possible health status of criteria (Table 2); where no health utility measures exist, our assumed health utilities are noted in Table 2. While health utility measures exist for (some) criteria, little is known about their relative importance against each other. Therefore, we used the “Analytic Hierarchy Process” [37–38] method to estimate the weight for each criterion against other criteria. This method provides a framework for ranking the criteria by calculated weights and for evaluating therapeutic alternatives. The weight for each criterion are determined by sequentially comparing the reciprocal of health utilities (Table 2) of all possible pairs of criteria and the eigenvectors [39] calculated in Analytic Hierarchy Process [33–35]. Specific (sub)criteria that are of higher concern to patients have higher values in weights. Based on this approach, the weights for “benefits” and “risks” criteria for the general population were 0.631 and 0.369 respectively. The weights for sub-criteria in descending order were prevention of ischemic stroke (0.43), risk of intracranial bleeding (0.252), prevention of systemic embolism (0.202), and risk of extracranial bleeding e.g., gastrointestinal bleeding (0.116).

**Table 2:** Health utilities inputted in models.

Criteria	Scenario	Criteria	Variables	Health Utility	Reciprocal of Health Untility	Reference
Benefit	General (70 year old)	Prevention of ischemic stroke	ischemic stroke	0.27 (0.22–0.32)	3.70 (3.13–4.55)	33
		Prevention of systemic embolism	systemic embolism	0.575 (0.45–0.7)	1.74 (1.43–2.22)	33
	High risk of stroke	Prevention of stroke or systemic embolism	stroke or systemic embolism	0.50 (0.22–0.70)	2.00 (1.43–4.55)	assumed
	Primary stoke prevention	Prevention of stroke or systemic embolism	stroke or systemic embolism	0.50 (0.22–0.70)	2.00 (1.43–4.55)	assumed
		Prevention of death from vascular causes	death from vascular causes	0.10	10.00	assumed
	Secondary stroke prevention	Prevention of stroke or systemic embolism	stroke or systemic embolism	0.50 (0.22–0.70)	2.00 (1.43–4.55)	assumed
		Prevention of death from vascular causes	death from vascular causes	0.10	10.00	assumed
Risk	General (70 year old)	Risk of intracranial bleeding	intracranial hemorrhage	0.46 (0.22–0.9)	2.17 (1.11–4.55)	33
		Risk of extracranial bleeding	extracranial hemorrhage	0.997 (0.98–1.00)	1.00 (1.00–1.02)	33
	High risk of stroke	Risk of major bleeding	major hemorrhage	0.80 (0.5–0.99)	1.25 (1.02–2.00)	35
	Primary stoke prevention	Risk of intracranial bleeding	intracranial bleeding	0.46 (0.22–0.9)	2.17 (1.11–4.55)	33
		Risk of other local bleeding	other local bleeding	0.997 (0.98–1.00)	1.00 (1.00–1.02)	33
	Secondary stroke prevention	Risk of intracranial bleeding	intracranial bleeding	0.46 (0.22–0.9)	2.17 (1.11–4.55)	33
		Risk of other local bleeding	other local bleeding	0.997 (0.98–1.00)	1.00 (1.00–1.02)	33

High risk of stroke: patients with CHADS_2_ score ≥3.

Finally, we calculated a performance score for each oral anticoagulant agent and warfarin that combined all of its sub-criteria of benefits and risks and their weights, relative to other agents; drugs with higher performance scores are preferred. Sensitivity analyses of weights (derived from the range of health utilities; Table 2) for major sub-criteria (prevention of ischemic stroke and risk of intracranial bleeding) were also conducted to determine how variations in the assigned weights for these criteria would affect the results.

## Results

### The general population (70 years old)

Table 3 presents the results of MCDA by a ranking matrix. Considering the overall benefit-risk, dabigatran 150 mg BID (0.529) had the highest performance score; other anticoagulants in descending order by performance scores were rivaroxaban 20 mg QD (0.462), apixaban 5 mg BID (0.426), and warfarin (0.191). On benefits alone, dabigatran 150 mg BID had the highest performance score (0.57); other anticoagulants in descending order based on performance scores were rivaroxaban 20 mg QD (0.506), apixaban 5 mg BID (0.393), and warfarin (0.284). On the basis of risks alone, anticoagulants in descending order by performance scores were apixaban 5 mg BID (0.483), dabigatran 150 mg BID (0.458), rivaroxaban 20 mg QD (0.386), and warfarin (0.030).

**Table 3:** Calculated performance scores and ranking.

				Weight	Warfarin	Dabigatran 150 mg BID	Dabigatran 110 mg BID	Rivaroxaban 20 mg QD	Apixaban 5 mg BID
**1. General (70 year old)**									
**Overall risk-benefit assessment**			Score	1	0.191	0.529		0.462	0.426
			Ranking		4	1		2	3
	Benefits		Score	0.631	0.284	0.57		0.506	0.393
			Ranking		4	1		2	3
		Prevention of ischemic stroke	Score	0.43	0.372	0.769		0.456	0.511
			Ranking		4	1		3	2
		Prevention of systemic embolism	Score	0.202	0.096	0.147		0.613	0.143
			Ranking		4	2		1	3
	Risks		Score	0.369	0.030	0.458		0.386	0.483
			Ranking		4	2		3	1
		Risk of intracranial bleeding	Score	0.252	0.000	0.639		0.230	0.598
			Ranking		4	1		3	2
		Risk of extracranial bleeding	Score	0.116	0.097	0.066		0.724	0.234
			Ranking		3	4		1	2
**2. High risk of stroke (CHADS2 score** ≥**3)**									
**Overall risk-benefit assessment**			Score	1	0.116	0.462	0.392	0.271	0.686
			Ranking		5	2	3	4	1
	Benefits: Prevention of stroke or systemic embolism		Score	0.615	0.120	0.697	0.535	0.372	0.748
			Ranking		5	2	3	4	1
	Risks: Risk of major bleeding		Score	0.385	0.109	0.085	0.165	0.109	0.586
			Ranking		3	5	2	3	1
**3. Primary stroke prevention**									
**Overall risk-benefit assessment**			Score	1		0.678	0.474	0.581	0.589
			Ranking			1	4	3	2
	Benefits		Score	0.791		0.753	0.468	0.642	0.629
			Ranking			1	4	2	3
		Prevention of stroke or systemic embolism	Score	0.132		0.758	0.383	0.575	0.503
			Ranking			1	4	2	3
		Prevention of death from vascular causes	Score	0.659		0.752	0.485	0.655	0.654
			Ranking			1	4	2	3
	Risks		Score	0.209		0.396	0.496	0.353	0.438
			Ranking			3	1	4	2
		Risk of intracranial bleeding	Score	0.143		0.393	0.525	0.194	0.377
			Ranking			2	1	4	3
		Risk of other local bleeding	Score	0.066		0.402	0.433	0.697	0.570
			Ranking			4	3	1	2
**4. Secondary stroke prevention**									
**Overall risk-benefit assessment**			Score	1		0.585	0.901	0.581	0.601
			Ranking			3	1	4	2
	Benefits		Score	0.791		0.616	0.923	0.642	0.614
			Ranking			2	1	4	3
		Prevention of stroke or systemic embolism	Score	0.132		0.739	0.652	0.575	0.731
			Ranking			1	3	4	2
		Prevention of death from vascular causes	Score	0.659		0.592	0.978	0.655	0.590
			Ranking			3	1	2	4
	Risks		Score	0.209		0.467	0.817	0.353	0.554
			Ranking			3	1	4	2
		Risk of intracranial bleeding	Score	0.143		0.594	1.017	0.194	0.656
			Ranking			3	1	4	2
		Risk of other local bleeding	Score	0.066		0.194	0.383	0.697	0.332
			Ranking			4	2	1	3

**Fig 2:**
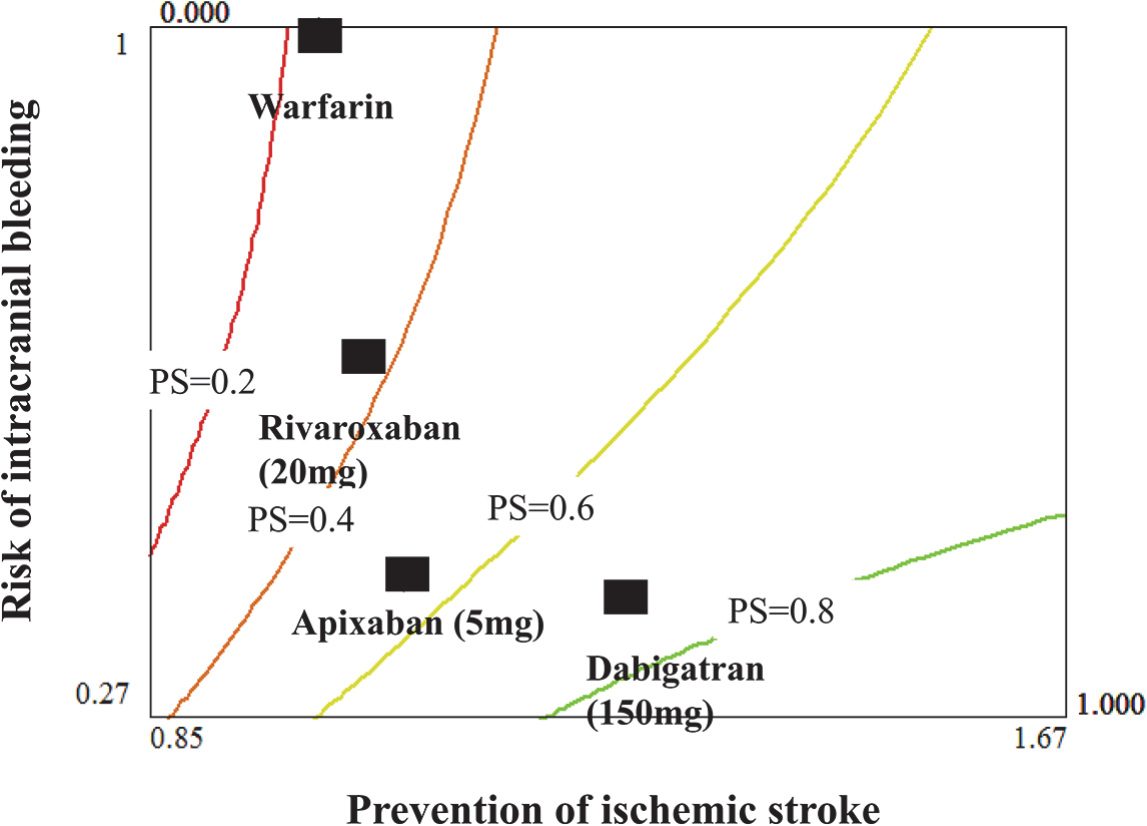
Iso-performance score curves for prevention of ischemic stroke and risk of intracranial bleeding.

Iso-performance score curves for prevention of ischemic stroke (reflected by the reciprocal of hazard ratio) and risk of intracranial bleeding (reflected by the hazard ratio) are shown in Fig 2. Each iso-performance score curve was calculated according to multi-attribute utility function principles [40]. For prevention of ischemic stroke and risk of intracranial bleeding, two major sub-criteria of concerns to physicians when selecting an anticoagulant agent, dabigatran 150 mg BID was the top priority for the general population because its performance score was closest to the highest iso-performance score curve (0.8). In contrast, warfarin and rivaroxaban were the least preferred choices; their performance scores were closest to the lowest iso-performance score curve (0.2).

### Patients with a higher risk of stroke

For patients with a high risk of stroke (CHADS_2_ score≥3), we found that apixaban had the highest performance score (0.686) considering both “benefits” and “risks” criteria. Other anticoagulants in descending order by performance scores were dabigatran 150 mg BID (0.462), dabigatran 110 mg BID (0.392), rivaroxaban 20 mg QD (0.271), and warfarin (0.116). When we focused on benefits only, apixaban 5 mg BID had the highest performance score (0.748); other anticoagulants in descending order based on performance scores were dabigatran 150 mg BID (0.697), dabigatran 110 mg BID (0.535), rivaroxaban 20 mg QD (0.372), and warfarin (0.12). When we focused on risks only, anticoagulants in descending order by performance scores were apixaban 5 mg BID (0.586), dabigatran 110 mg BID (0.165), rivaroxaban 20 mg QD (0.109), warfarin (0.109) and dabigatran 150 mg BID (0.085).

### Primary and secondary prevention

For primary stroke prevention, we only compared new oral anticoagulants. In the overall benefit-risk assessment, anticoagulants in descending order by performance scores were dabigatran 150 mg BID (0.678), apixaban 5 mg BID (0.589), rivaroxaban 20 mg QD (0.581), and dabigatran 110 mg BID (0.474). The order was similar when we focused on benefits only: dabigatran 150 mg BID (0.753), rivaroxaban 20 mg QD (0.642), apixaban 5 mg BID (0.629), and dabigatran 110 mg BID (0.468).

For secondary stroke prevention, anticoagulants in descending order by performance scores were dabigatran 110 mg BID (0.901), apixaban 5 mg BID (0.601), dabigatran 150 mg BID (0.585), and rivaroxaban 20 mg QD (0.581) in the overall benefit-risk assessment. The order was the same when we assessed risks only; based on performance scores: dabigatran 110 mg BID (0.817), apixaban 5 mg BID (0.554), dabigatran 150 mg BID (0.467), and rivaroxaban 20 mg QD (0.353).

**Fig 3:**
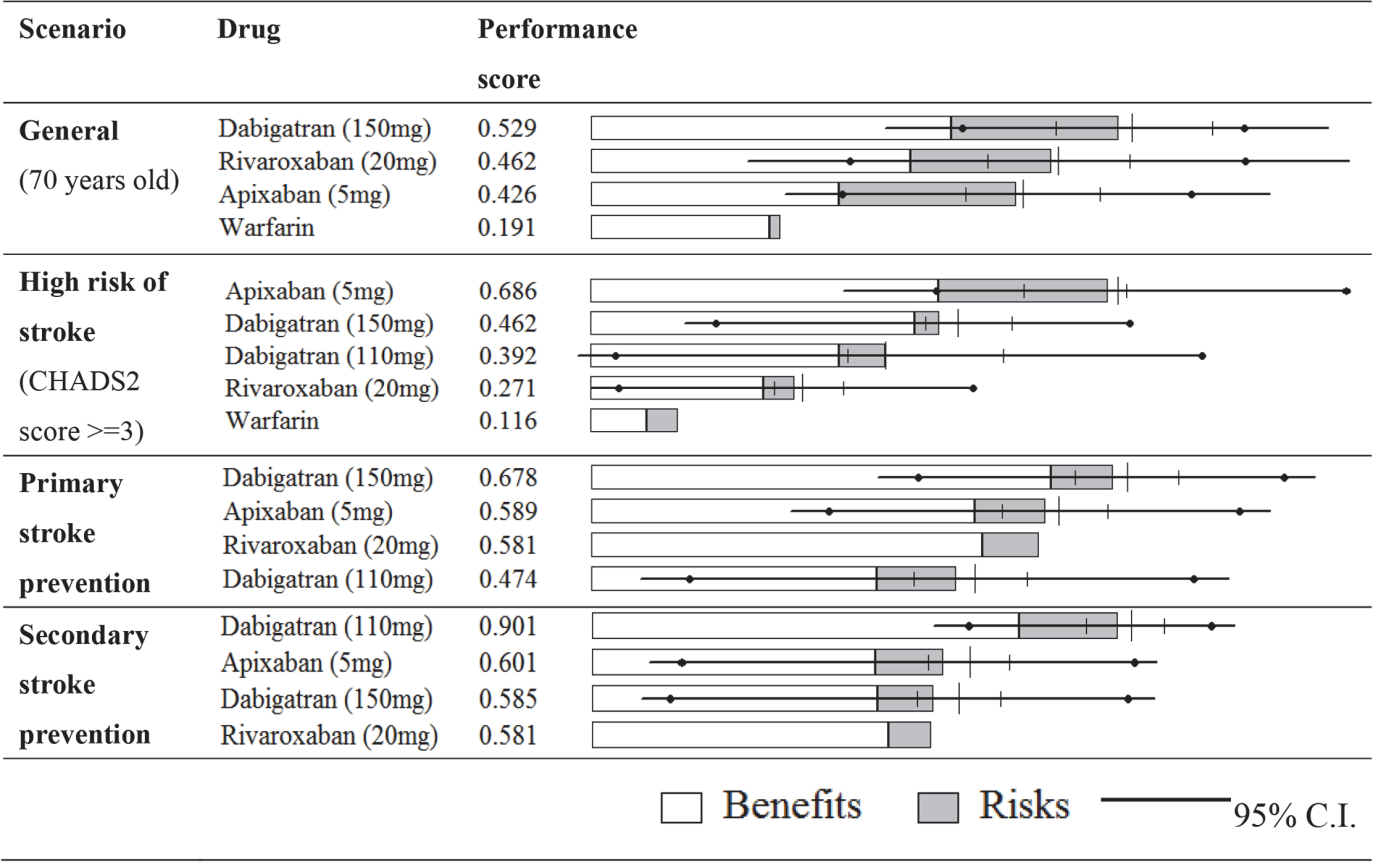
Ranking of oral anticoagulants by performance scores under various scenarios.

Fig 3 showed the ranking of oral anticoagulants under various scenarios by histograms. It not only indicated the ranking of overall performance scores for each alternative, but also how “benefits” and “risks” contributed toward performance scores. In Fig 3, the 95% confidence intervals of hazard ratios reflected the minimum and maximum values in our models to represent uncertainties.

### Sensitivity Analysis

**Fig 4:**
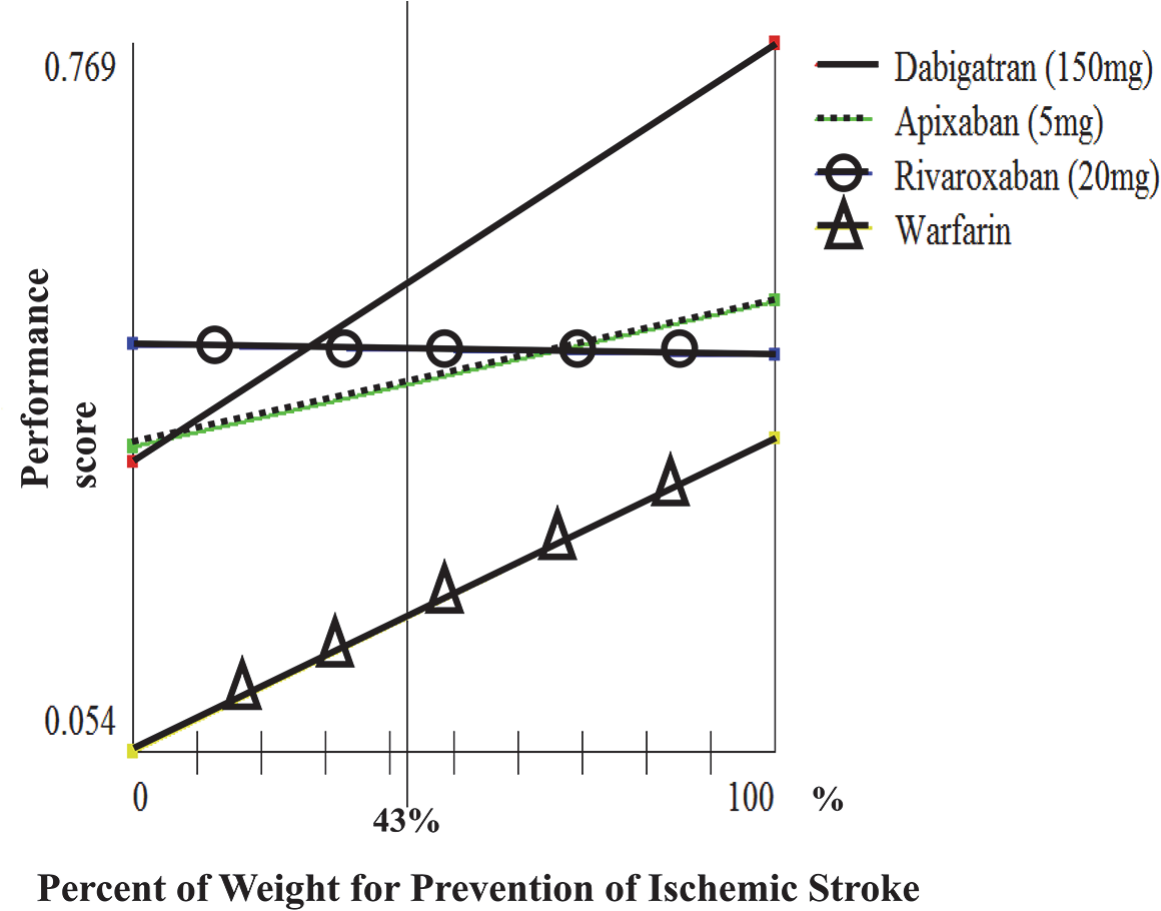
Sensitivity of weight for prevention of ischemic stroke.

**Fig 5:**
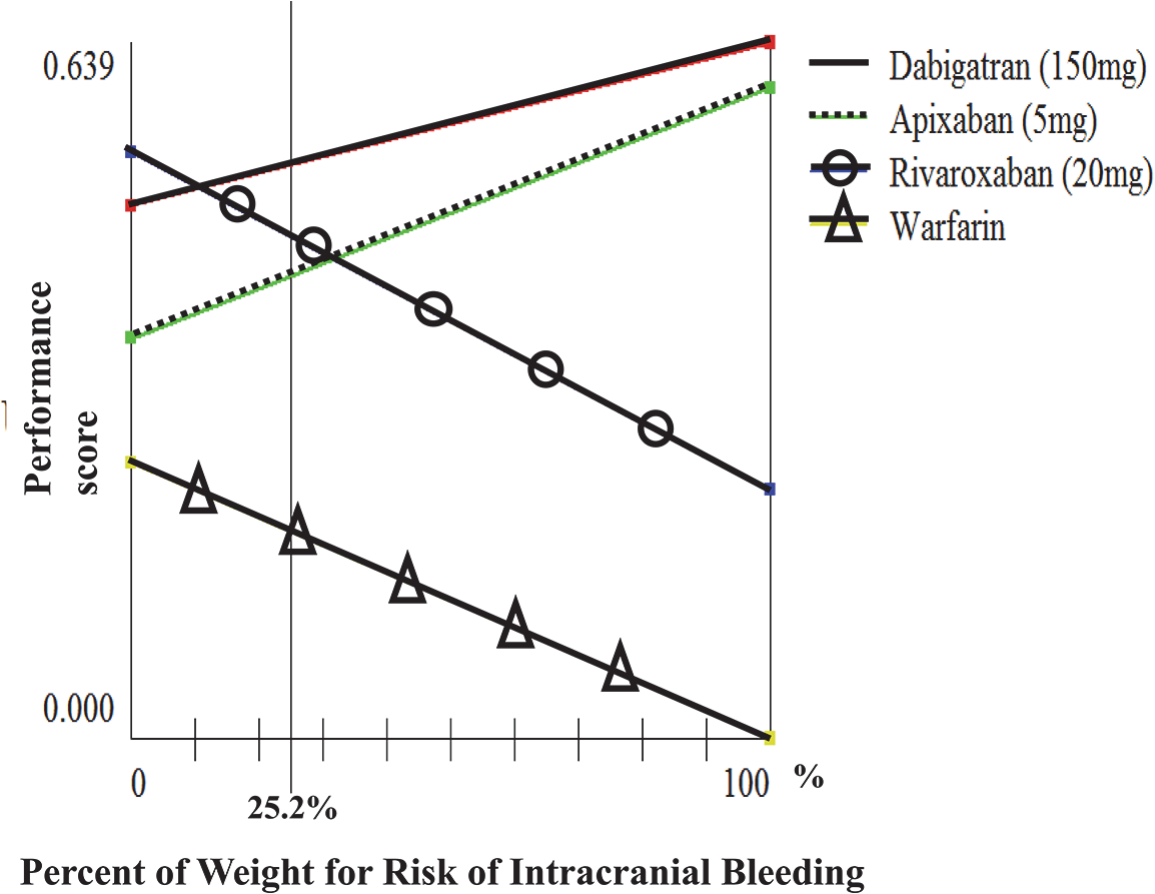
Sensitivity of weight for risk of intracranial bleeding.

We conducted sensitivity analyses of weights for prevention of ischemic stroke (which has the highest weight among “benefits” sub-criteria) and risk of intracranial bleeding (which has the highest weight among “risks” sub-criteria) on performance scores for the general population. We found that if the weight for prevention of ischemic stroke was between 0.05 and 0.65, dabigatran 150 mg BID and rivaroxaban 20 mg QD had the highest performance scores (Fig 4). Also, dabigatran 150 mg BID and rivaroxaban 20 mg QD were the optimal choices when the weight for prevention of intracranial bleeding was less than 0.32 (Fig 5).)

## Discussion

This is the first study to compare the benefit-risk balance of oral anticoagulants in patients with nonvalvular atrial fibrillation through MCDA with consideration of the relative importance of different clinical outcomes from patients’ perspectives. The MCDA method can compare more than two drugs; it can compare the effects of multiple drugs on the same outcome (clinical endpoints to reflect efficacy and/or risks); and it can also “conjointly” evaluate the comparative efficacy and safety of multiple drugs, considering their benefits, risks, and the benefit-risk balance at the same time [25–26]. Our results indicate that among new oral anticoagulants, dabigatran 150 mg BID, compared with rivaroxaban 20 mg QD and apixaban 5 mg BID, might be the first choice for patients with AF in the overall risk-benefit assessment. When we consider specifically prevention of ischemic stroke and risk of intracranial bleeding, dabigatran 150 mg BID still has high priority. Our sensitivity analyses also indicate that dabigatran 150 mg BID is either first or second choice if the weights for prevention of ischemic stroke and risk of intracranial bleeding are within certain ranges.

Our analysis aims to provide an integrated evaluation of risks and benefits; thus, cost was not included. Our results differ with the findings by Canestaro et al, which was a cost-effectiveness analysis. Canestaro et al found that apixaban 5 mg BID is the optimal choice, it being the most cost-effective strategy, and warfarin is the best option in probabilistic sensitivity analysis [24].

Our analyses show the differences between anticoagulants in relation to specific outcomes of benefits and risks. Such risk-benefit-risk assessment of drugs can help to guide clinicians in making treatment decisions for individual patients. For instance, while dabigatran 150 mg BID is the best choice in the overall benefit-risk assessment, its benefit is small for prevention of systemic embolism, and in fact, its performance score is the lowest among all drugs when the risk of extracranial bleeding is the main consideration. In this case, dabigatran 150 mg BID is still not the suggested choice for patients with a high risk of extracranial bleeding (especially for older patients), even though it has the highest performance score in our overall benefit-risk assessment.

For patients with a higher risk of stroke (CHADS_2_ score≥3), our results indicate that apixaban 5 mg BID is the first choice, and it is substantially better than the next alternative by performance scores (0.686 vs. 0.462 for dabigatran 150 mg BID). This result is similar to Schneeweiss et al [16], who compared efficacy and safety of all agents based on data from several large clinical trials. Schneeweiss et al also proposed that apixaban is the best mainly due to statistically similar rates of stroke and systemic embolism but a much lower risk of major bleeding compared with other drugs. Our study also shows the differences between drugs toward other clinical endpoints using an integrated risk-benefit assessment, which provides helpful information to clinicians and patients.

Our findings are also similar to the indirect comparison analysis by Rasmussen et al [15] Rasmussen et al found that for primary stroke prevention, new oral anticoagulants showed some differences in efficacy and risk of bleeding, but no drug is significantly better than the other based on their balance of benefits and risks. For secondary stroke prevention, dabigatran 110 mg BID seems to be safer than other drugs. Our MCDA analysis found that dabigatran 150 mg BID is the first priority for primary stroke prevention; other drugs have similar performance scores. For secondary stroke prevention, dabigatran 110 mg BID is the priority; it is substantially better than the next choice (apixaban 5 mg BID) by performance scores.

This study has important clinical and policy implications. Despite great efforts made on comparative effectiveness research of new drugs, little attention has been paid to quantitative methods for integrated, comparative assessments of benefit-risk balances of drugs. Previous studies only compared drugs’ effects on single clinical outcomes one at a time [15–16]. In contrast, our study compared drugs’ effects on multiple clinical outcomes at once and additionally our method integrates benefits and risks. Our study provides an example of how benefit-risk balance of alternative drugs can be compared in an integrated fashion using available evidence. We also compared these agents under different scenarios, including population with a high risk of stroke and for primary and secondary stroke prevention. Integrated assessments of benefit-risk of medicines are valuable for clinical practice.

In spite of its findings and contributions, this study has several limitations. First, the present results only considered pharmacological options for patients with AF, and provided suggestions for choosing appropriate medications in clinical practice. This study did not consider “the necessity of taking new oral anticoagulants for the individual patient” (e.g., the probability of stroke or bleeding) or patient’s disease history. New anticoagulants should be used in patients without serious renal/liver/heart insufficiency for prevention of stroke. Second, most decision models including our models are a simplification of decision making when treating real patients. Our models considered specific clinical endpoints for measuring “benefits” and “risks” (e.g., intracranial bleeding) rather than broadly defined outcomes (e.g., bleeding) providing more detailed benefit-risk assessment of oral anticoagulants; however, outcomes can be further stratified, for example, using specific endpoints such as risk of gastrointestinal bleeding rather than including it in the category of local bleeding. We also did not consider medications’ properties such as interaction with other drugs, adherence rate, risk of switching medications, off-label or inappropriate use leading to adverse events and so on. Third, how to determine the suitable weights for each criterion with consideration of physicians’ and patients’ views has been a major challenge in MCDA modeling. While patient preferences need to be considered, these are not well studied to date. Thus, we adopted Analytic Hierarchy Process to estimate the weight for each criterion against other criteria based on existing health utility measures and conducted sensitivity analyses of weights to consider uncertainties, although these may not be particularly sensitive to individual patient preferences and trade-offs between different outcomes. Fourth, this study focused on four scenarios (general, high risk of stroke, primary and secondary stroke prevention), our findings and recommendations may not apply to other scenarios (e.g., patients without AF or patients >80 years old). Further research is therefore warranted to assess the benefit-risk of anticoagulants under different scenarios. Finally, drugs may have accumulated benefits and risks; however, time-dependant nature of risk and benefit was not considered in our MCDA models. Research is needed to advance MCDA method to enable consideration of time-dependant risks and benefits.

## Conclusion

To reduce the social and economic burdens of stroke, the most effective way is to prevent its occurrence. In addition to controlling modifiable risk factors associated with stroke, anticoagulant drugs play an important role for the prevention of stroke. However, clinicians and patients often face the challenge of making decisions between multiple treatment choices without integrated, comparative benefit-risk evidence. Using the MCDA quantitative method, this study evaluated the integrated efficacy and safety of oral anticoagulants and compared five treatment options under different scenarios. Our analysis presents a useful meta-analytic approach that allows studies of different types to be synthesized for an integrated assessment of the comparative risk-benefit balance of several drugs at once. Such evidence helps clinicians and patients to make an appropriate treatment decision to prevent stroke under the safer condition.
